# Natural Killer Cell Dysfunction and Its Role in COVID-19

**DOI:** 10.3390/ijms21176351

**Published:** 2020-09-01

**Authors:** Charmaine van Eeden, Lamia Khan, Mohammed S. Osman, Jan Willem Cohen Tervaert

**Affiliations:** Department of Medicine, Faculty of Medicine and Dentistry, University of Alberta, Edmonton, AB T6G 2R3, Canada; vaneeden@ualberta.ca (C.v.E.); lkhan@ualberta.ca (L.K.); mosman@ualberta.ca (M.S.O.)

**Keywords:** COVID-19, SARS-CoV-2, natural killer cells, immune dysregulation, cytokine storm

## Abstract

When facing an acute viral infection, our immune systems need to function with finite precision to enable the elimination of the pathogen, whilst protecting our bodies from immune-related damage. In many instances however this “perfect balance” is not achieved, factors such as ageing, cancer, autoimmunity and cardiovascular disease all skew the immune response which is then further distorted by viral infection. In SARS-CoV-2, although the vast majority of COVID-19 cases are mild, as of 24 August 2020, over 800,000 people have died, many from the severe inflammatory cytokine release resulting in extreme clinical manifestations such as acute respiratory distress syndrome (ARDS) and hemophagocytic lymphohistiocytosis (HLH). Severe complications are more common in elderly patients and patients with cardiovascular diseases. Natural killer (NK) cells play a critical role in modulating the immune response and in both of these patient groups, NK cell effector functions are blunted. Preliminary studies in COVID-19 patients with severe disease suggests a reduction in NK cell number and function, resulting in decreased clearance of infected and activated cells, and unchecked elevation of tissue-damaging inflammation markers. SARS-CoV-2 infection skews the immune response towards an overwhelmingly inflammatory phenotype. Restoration of NK cell effector functions has the potential to correct the delicate immune balance required to effectively overcome SARS-CoV-2 infection.

## 1. Natural Killer Cell Role in Immune Regulation

Natural killer (NK) cells form part of the innate immune system, where they serve as a first-line defense against acute infection and cancer, whilst also regulating the adaptive immune response [1]. NK cell function is tightly regulated by a balance of activating and inhibitory germline-encoded receptors. NK cell activation results in cytotoxic degranulation and the production of inflammatory cytokines, killing target cells [1,2,3,4]. Healthy cells express major histocompatibility complex class I (MHC I) molecules which mark these cells as “self”, MHC I act as ligands for inhibitory receptors on NK cells and contribute to the “self-tolerance”, by preventing NK-cell-killing of these cells [2,3]. The MHC I—specific inhibitory receptors include the killer cell immunoglobulin-like receptors (KIRs) (KLRG1, and TIGIT) and the lectin-like CD94-NKG2A heterodimers [2,5].

Cellular stress, impaired KIR engagement and MHC 1 downregulation, associated with infection or cancer growth, lower the inhibitory signalling threshold, resulting in NK cell activity receptor upregulation [2,4,5]. NK cells express numerous activating receptors, which in response to infection or cellular distress, induce signalling pathways (NKG2D, CD244, NKp30, NKp46) that trigger NK cell responses [2,3,4]. Through co-activation these receptors overcome the NK regulatory balance to mount an effective response [2,6]. Activated NK cells induce killing through multiple mechanisms; (1) NK cell activation can result in direct lysis of target cells, through cytotoxic degranulation by perforin and granzymeB, (2) indirect elimination of target cells through the production of inflammatory cytokines, such as interferon-γ (IFN-γ) and tumor necrosis factor-α (TNF-α), (3) NK cells express CD16, which allows for the detection of antibody-coated target cells, leading to NK cell antibody-dependent cell cytotoxicity (ADCC) and (4) through interaction with accessory cells such as monocytes, NK cells may indirectly also interact with infectious ‘nonself’ and Toll-like receptor (TLR) ligands, inducing IFNγ production and enhancing cytotoxicity [1,2,4,7]. NK function can also be downregulated, this is achieved through ligand interaction with inhibitory receptors such as killer immunoglobulin-like receptors (KIRs) and the C-type lectin-like receptor CD94-NKG2A which suppress NK cell activation [1,2].

**Figure 1:**
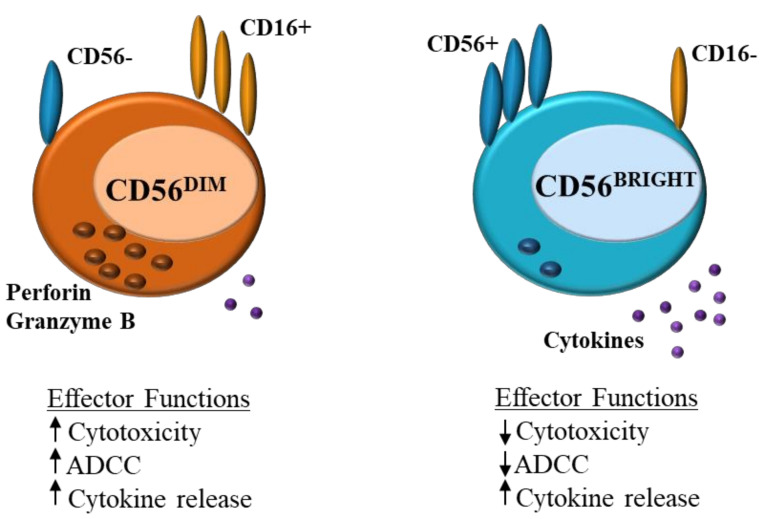
Natural killer (NK) cells: differences in effector function between CD56^DIM^ and CD56^BRIGHT^ ADCC = antibody-dependent cell cytotoxicity.

NK cells can be divided into CD56^DIM^ and CD56^BRIGHT^ subsets (Figure 1). CD56^DIM^CD16^+^ NK cells are abundant in the blood and are cytotoxic, expressing perforin and producing IFN-γ, CD56^BRIGHT^CD16^−^ cells on the other hand are found in lymphoid tissues, these cells lack perforin activity and instead produce cytokines such as IFN-γ in response to stimulation with IL-12, IL-15 and IL-18, increasing NK effector function [4,5,8].

Apart from their critical role in pathogen elimination, an equally important role of NK cells is their potential to limit the immune response, specifically T cells. Indirectly this is achieved through the modulation of antigen presenting cells (e.g., dendritic cells (DCs)) and directly through interactions with the T cells themselves (1, 2). During acute infection, NK cells can promote the differentiation of naïve CD4+ T cells into Th1 T cells through the secretion of IFNγ, thereby leading to increased pathogen control, they can also decrease T cell priming through IL-10 (1, 2). NK cell promotion of mature DCs leads to increased antigen presentation and thus an increased CD8+ T cell response (1, 2, 4). Otherwise, NK cells also regulate the adaptive immune response through cytotoxic killing of activated T cells. Activated T cells often express higher levels of activating ligands (NKG2D) and decreased inhibitory ligands (MHC I), resulting in their recognition and elimination (2, 3). Resultant reduction in T follicular helper (Tfh) responses hampers the development of the B cell response, as Tfh are responsible for driving B cell differentiation into memory B cells and plasma cells that produce antibodies [9].

## 2. Unbalanced Immune Responses in Autoimmune and Systemic Conditions

There are many parallels between infection, genetics and physiology and NK cell dysregulation. In this section we share aspects of NK cell dysfunction that are not related to infections agents.

### 2.1. Age and Sex Related Immunoscenescence

Ageing is associated with the development of chronic inflammation and a general reduction in immune diversity [10]. The aged innate immune response is characterised by the increased secretion of pro-inflammatory cytokines (TNF, IL-6, and IL-1β) and a decrease in the number and function of DCs and macrophages, leading to poor priming of T cells and diminished clearance of infectious agents and apoptotic cells through phagocytosis [11,12]. Cell-mediated immunity suffers a loss in naïve lymphocytes, with an increased expansion of antigen-specific memory lymphocytes, leading to an inadequate immune response to newly encountered antigens and an increased susceptibility to infection [11,12,13]. NK cells as mediators of immune regulation play a pivotal role in this immune senescence (Figure 1).

NK cell production and proliferation are reduced in ageing, though the absolute number of NK cells is higher, likely due to the accumulation of long-lived NK cells [14]. Expression of NCR NKp30 is reduced in ageing, this not only decreases granule-mediated cytotoxicity but also negatively impacts the adaptive immune response through obstructed NK-DC crosstalk [15]. Neutrophil apoptosis by NK cells is mediated through death receptor ligation by NCR NKp46. Ageing results in a reduction of neutrophil apoptosis and an increase in neutrophil necrosis, which induces inflammatory responses and local tissue damage [16]. Both NKp30+ and NKp46+ NK cell expression is reduced in older individuals (Table 1) [17].

Composition of NK cell subsets in older adults shows a higher frequency of mature NK cells (CD57^+^), and an increased CD56^DIM^:CD56^BRIGHT^ ratio. The increase in CD56^DIM^ cells may be compensatory to the loss of natural killer cell cytotoxicity observed in older individuals [17]. Reduced cytotoxicity is associated with impaired immune regulation through the accumulation of senescent cells and a reduced lysis of DC, CD4^+^ and CD8^+^ cells [18]. Decline in CD56^BRIGHT^ NK cells leads to reduced immune regulatory capacity through decreased IFNy, MIP-1a and IL-8 production [15].

Sex also influences the humoral and cell-mediated immune responses. In young adults females often have a more robust response, with T and B cell populations being higher than those in males, NK cell activity is however higher in men [11,19]. In contrast, in women with menopause the number of T and B cells are reduced and NK cell cytotoxicity is increased [11].

### 2.2. Atherosclerosis and Cardiovascular Disease

**Figure 2:**
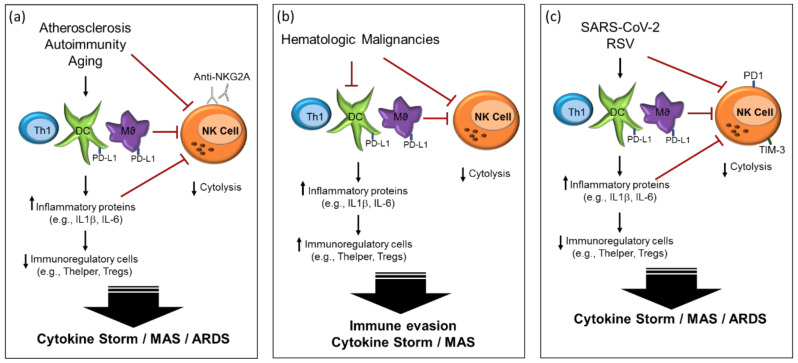
Natural killer cell dysregulation in (**a**) systemic diseases; (**b**) hematologic malignancies; (**c**) SARS-CoV-2. (**a**) Immune imbalance in atherosclerosis, autoimmunity and ageing leads to excess inflammation; (**b**) Hematologic malignancies trigger both excess inflammation and immune invasion; (**c**) SARS-CoV-2 and respiratory syncytial virus (RSV) trigger immune imbalances which result in a disproportionate inflammatory response.

Atherosclerosis is characterised by vascular inflammation and is one of the leading causes of morbidity and mortality arising from coronary artery disease, stroke and peripheral vascular disease. The disease course is characterised by an abundance of monocyte-derived macrophages. Accumulated low density lipoproteins (LDL) undergo oxidative modification, facilitating the uptake of oxidized LDL by macrophages. Activated macrophages in turn generate inflammatory cytokines and chemokines that promote inflammation and contribute to the regulation of monocyte and T cell infiltration (Figure 2) [36]. Pathogenic T cells in atherosclerosis have the characteristics of Th1, generating pro-inflammatory cytokines such as IFN-γ and performing the activation of macrophages. Additionally, oxidised phospholipids (oxLDL) trigger inflammation, through TLR binding.

Atherosclerotic plaques (AP) are in general stable and unlikely to produce symptoms, however when these plaques become unstable they have the potential to increased vascular complications. The CD56^BRIGHT^ NK cell subset has been shown to be increased in AP, particularly in symptomatic patients, suggesting their preferential accumulation in unstable plaques [20]. Furthermore, studies have also revealed expression of MICA/B in AP. MICA/B serve as ligands for the NK activating receptor NKG2D [20,21]. Together this suggests NK cells contribute to tissue damage and inflammation in atherosclerosis, through increased cytokine production and NK cell lysis. Additionally, chronic cytomegalovirus (CMV) infection strongly activates NK cells, characterised by an increase in the NK cell activating receptor NKG2C, resulting in increased inflammation and the exasperation of atherosclerotic symptoms (Table 1) [37,38].

### 2.3. Autoimmunity

Although autoimmune diseases are largely characterised as being associated with T and B cell lymphocytes, NK cells appear to have a role both in the stimulation of these altered adaptive immune responses and in self-tolerance mechanisms (Table 1) [39].

Systemic lupus erythematosus (SLE) is a chronic autoimmune disease characterised by the presence of autoantibodies, immune dysregulation and damage to kidney, skin, heart and lung tissues. In SLE apoptosis is either increased or clearance suboptimal, leading to an increase in autoantigen-antibody complexes stimulating interferon alpha (IFN- α). Increased expression of IFN- α activates lymphocyte, DCs and natural killer (NK) cells, and leads to the upregulation of several inflammatory proteins [40]. Patients with SLE have a numerical deficit and a reduced cytotoxicity of NK cells. In addition, Sederberg et al., demonstrated that SLE patients may foster autoantibodies to both HLA class I-binding receptors (NKG2A, NKG2C), and multiple killer cell immunoglobulin-like receptors, which results in the dysregulation of self-nonself-recognition [22,25] (Table 1).

Juvenile idiopathic arthritis (JIA) presents as chronic synovitis and is the most common cause of chronic arthritis in children [41]. Systemic JIA patients have profoundly diminished NK cell cytolytic activity and a proportion of patients also have reduced numbers of circulating CD56^BRIGHT^ NK cells [26]. This pattern of NK cell dysfunction, mirrors that of macrophage activation syndrome (MAS) (described in Section 2.5), and indeed, MAS has been described in around 10% of JIA patients [42].

Multiple sclerosis (MS) is characterised by demyelination involving the central nervous system, which leads to a progressive cognitive decline and physical disability [43]. MS clinical relapse has been shown to be associated with decreased circulating NK cells [23], additionally a higher ratio of CD56^BRIGHT^/CD56^DIM^ NK cells has been found in cerebrospinal fluid (CSF) of MS patients with respect to controls [24]. This reduction in cytotoxic NK cells is associated with defective regulation of T-cell activity, resulting in CD4^+^ T cell evasion due to impaired DNAM-1 (DNAX accessory molecule-1) interactions [44].

### 2.4. Hematologic Malignancies

Hematologic malignancies include leukemia, chronic myeloid neoplasms, B-, T- and NK cell lymphomas, as well as multiple myeloma. In general, cancers evade the host immune system through mechanisms of apoptotic resistance and immune cell deactivation. Specifically, however, hematological malignancies result in metabolic changes that directly suppress effector immune responses [27,29,45,46,47]. Rapidly dividing cancer cells increase the expression of immune inhibitory molecules which skew the balance of immune activation to promote immune dysregulation. One of these mechanisms involves loss of surface HLA class I and II as well as CD58 expression and the upregulation of molecules such as Galectin-9, CD274 and CD47 which provide cancer cells with direct survival signals, but they also inhibit infiltrating immune cells from clearing apoptotic signals or from executing cytolytic pathways [27,45,46,47] (Table 1). Though the loss of HLAI and HLAII expression would usually lead to increased NK cell cytolytic activity, the additional loss of CD58 prevents NK cell activation [27,45].

Moreover, hematological malignancies may release other immune suppressive signals such as adenosine and IL-10. Extracellular adenosine (ADO), indirectly inhibits the maturation, cytotoxicity and effector function of NK cells, through modification of specific cellular pathways [48]. Increased expression of IL-10 inhibits DC priming, and promotes Th2 and Treg cell differentiation, increased TGFβ also promotes Treg differentiation, and indoleamine 2,3-dioxygenase (IDO) suppresses CTL and NK immune responses through degradation of tryptophan [27]. A reduction in the expression of CD58 results in reduced activation of NK and cytotoxic T cells (Figure 2) [45]. This combined downregulation of activated NK cells leads to cancer escape, allowing for tumor growth and spread.

Aggressive natural killer leukemia (ANKL) is a rare systemic proliferation of mature NK cells largely associated with Epstein Barr virus (EBV) [28]. ANKL is characterised by CD2^+^, CD16^+^ and CD56^+^ cells positive for cytotoxic molecules. High serum levels of CXCR1 indicated increased CD56^DIM^ NK cell activity resulting in necrosis, apoptosis and organ failure [49]. In stark contrast to the expansion of NK cells in ANKL, syndromes characterised by severely diminished NK cell numbers and NK cell subsets also exist. Classical natural killer cell deficiency (CNKD) leaves patients highly susceptible to viral infection. Genetic mutations result in not only a decrease in NK cell numbers but also in a loss of the CD56^BRIGHT^ NK cell subset, the CD56^DIM^ cells, if present are characterised by functional impairment [50], together resulting in collapse of the innate immune response. Impaired NK cell function is also observed in various primary immune deficiencies in particular, in familial hemophagocytic lymphohistiocytosis (HLH, discussed in Section 2.5), NK cell cytolytic activity is significantly reduced or even abrogated [51].

### 2.5. Hemophagocytic Lymphohistiocytosis

Hemophagocytic lymphohistiocytosis (HLH) is a rare, life-threatening disease associated with an overwhelming systemic immune activation. HLH can be either genetic (familial or immunodeficiency syndrome) or acquired, the latter being associated with viral infection, malignancies and autoimmune disease [30]. The most prominent immunological feature of patients with genetic or acquired HLH is the loss of natural killer (NK) cell effector functions [52,53,54]. Familial hemophagocytic lymphohistiocytis (FHL) is an autosomal recessive disease caused by several gene mutations that participate in the granule-dependent cytotoxic function of NK and T cells [55]. The syndrome is characterised by high ferritin levels, increased levels of pro-inflammatory cytokines (IFN-γ, TNFα, IL-6, IL-8, IL-10, IL-12, IL-18 and MIP-1α), persistent activation of macrophages and T cells and systemic inflammation.

Acquired HLH is most commonly associated with haematological malignancies, such as T cell and NK cell leukaemia [56] and has been identified in patients undergoing chemotherapy. In adults, viral infection plays a leading role in the development of acquired HLH [57], with herpes viruses being the most prominent triggers (Epstein-Barr virus (EBV), cytomegalovirus (CMV)). Influenza, Dengue and Ebola viruses have also been recognised, along with human immunodeficiency virus (HIV). In rheumatic diseases HLH is termed macrophage activation syndrome (MAS), where viral infections are a recognised trigger [42]. Similarly to familial HLH, patients with systemic juvenile idiopathic arthritis (sJIA) harbor mutations in genes related to NK cell cytotoxicity [58,59]. MAS also occurs in adult onset Still’s disease [60] and systemic lupus erythematosus (SLE) [25], where in the latter autoantibodies targeting CD94^+^, result in an increase in NK cell cytokine secretion [22].

## 3. Viral Infection Immune Dysregulation

### 3.1. Pandemic 2009 (H1N1) Influenza A (Pandemic A(H1N1) 2009)

The 2009 influenza pandemic occurred between early 2009 and middle 2010, and it is estimated that between 700 million to 1.4 billion people were infected and that between 150,000 and 410,000 deaths may have occurred [61]. Pandemic A(H1N1) 2009 was more likely to affect younger individuals (<65) and those with comorbidities. Symptoms in general were mild (fever, cough, malaise), but a small percentage of individuals developed more severe disease such as pneumonia, myocarditis and ARDS [61,62].

Pandemic A(H1N1) 2009 infects DCs, macrophages, and NK cells through cell surface sialic acids, where it fails to effectively activate IFNγ and TNFα gene expression, and induces only minimal antiviral and pro-inflammatory cytokines, allowing for potential immune escape. Disease severity has been correlated to an increase in the inflammatory markers IL-6, IL-10, IL-15 and MCP-1 [63]. Influenza viral proteins are recognised by NK cell NCR NKp46, leading to lysis-infected targets and the production of cytokines, the viruses have however developed the ability to alter glycosylation of these proteins, limiting NKp46 interactions and allowing for potential immune escape [64]. Influenza virus has also been shown to redistribute MHC 1 proteins for enhanced interaction with inhibitory receptors, promoting viral survival [64,65]. Alterations of the adaptive immune responses are predominated by increase of Tregs, decreased levels of CD3+, CD4+ and CD8+ cells, as well as an increased CD4+/CD8+ ratio [63,66,67]. The reduction of cytolytic activities (NK, CD8+ T cells) and the increase of Tregs facilitates the survival of Pandemic A(H1N1) 2009 infected cells and promotes inflammation and tissue damage. HLH was rarely reported in Pandemic A(H1N1) 2009 [68].

### 3.2. Severe Acute Respiratory Syndrome (SARS)

Severe acute respiratory syndrome first emerged in China in November 2002, where after it rapidly spread across the world, 8096 infections were reported with a mortality rate of 9.6% [69]. SARS is caused by the RNA coronavirus SARS-CoV and is characterised by severe clinical manifestations of the lower respiratory tract [69]. The pathology of SARS is however related to dysregulation of the immune response, which is highlighted by the involvement of the spleen, lymph nodes and circulating lymphocytes [70]. Angiotensin-converting enzyme 2 (ACE2) has been identified as a SARS-CoV receptor and tissue expression of the receptor correlates with the localization of the virus [71,72]. Binding of the SARS-CoV proteins to ACE2 reduces its expression, promoting disease pathogenesis by inducing edema and impaired lung function [71,72]. Cellular infiltration by activated macrophages has been observed in the lungs of SARS patients [71].

CD4^+^, CD8^+^, CD20^+^, DC, macrophage and NK cell populations are all decreased in SARS patients. In addition to reduced numbers of NK cells, a reduction in immunoglobulin-like receptor CD158b+ NK cells has also been observed [73], suggesting that cytotoxicity is decreased. SARS-CoV has been shown to infect both T lymphocytes and monocytes, contributing to lymphopenia (HLH reported in some patients), loss of germinal centres and the destruction of spleen and lymphoid tissues [69,74]. Elevated chemokine (IL-8, MCP1 and IP-10) and cytokine (IL-1β, IL-6) levels may be linked to an overactive innate immune response, leading to the recruitment of macrophages, and the activation of cell-mediated immunity [69,75,76].

### 3.3. Respiratory Syncytial Virus (RSV)

Respiratory syncytial virus (RSV) is a major cause of severe lower respiratory tract disease, and is the leading cause for mortality in infants and the elderly [32,77]. Infection is characterised by airway obstruction, runny nose, shortness of breath, wheezing, hypoxia and in severe cases, pneumonia and bronchiolitis [78]. There is also an association with the development of asthma in infants at an early age [78]. At birth, neonates, rely on maternally derived antibodies and innate responses to defend against pathogens. Furthermore, RSV-susceptibility increases between the ages of 2 and 6 months when maternal antibodies levels have decreased [79].

Severe RSV disease is characterised by a skewing of the immune response towards a dysregulated Th2-type response, where the production of IFN-I is inhibited, decreasing the Th1 antiviral response [80,81]. RSV infects NK cells, and although this does not result in the release of infectious particles, the expression of receptors is altered leading to a shift in effector function [31,77]. NK cells infected with RSV express higher levels of IFN-γ, as well as the NKG2D ligand MICA and reduced expression of activating receptors NKG2D and NKp44 [77,78]. RSV thus skews NK cells towards a more inflammatory phenotype resulting in exacerbation of disease symptoms.

## 4. Immunological Features of COVID-19

SARS-CoV-2 is the causal agent of coronavirus disease 2019 (COVID-19). This ongoing pandemic has infected over 1.7 million people and resulted in over 800,000 deaths [82,83]. SARS-CoV-2 [34,35,84] infection in most cases is characterised by mild symptoms of fever, tiredness and dry cough. In rare cases however severe or even fatal disease develops which is brought on by severe inflammatory signaling resulting in a cytokine release syndrome ranging from pyrexia to severe clinical manifestations such as hemophagocytic lymphohistiocytosis (HLH) and acute respiratory distress syndrome (ARDS) [34,35,84,85,86,87]. Severe complications associated with infection are more common in the elderly particularly those with cardiovascular diseases [86,88].

The immunopathology of COVID-19, as was the case for SARS, is based on the dysregulation of the innate and cell-mediated immune responses. SARS-CoV-2 has also been shown to utilize ACE2 as a cell entry receptor [89,90], intriguingly ACE2 activity is increased in both pulmonary and cardiovascular disease [91,92]. Multiple studies have found that patients infected with SARS-CoV-2 have significantly decreased numbers of NK and CD8+ T cells, and that these cells displayed a functionally exhausted phenotype [35,93,94], increased expression of the NK inhibitory marker NKG2A and T cell exhaustion markers PD-1 and Tim-3 [35]. These findings were reciprocated in other studies [84,85,87,95], which additionally highlighted excessive T cell activation marked by high levels of HLA-DR and CD38. In NK cells, expression of the inhibitory marker NKG2A leads to decreased expression of IFNγ, IL-2 and TNFα as well as reduced granzyme B levels. Tim-3, in contrast to its senescent role in T cells, has been suggested to be a marker of fully functional NK cells, resulting in increased IFNγ expression [33,96]. Tim-3 has however also been linked to the restraint of NK cell cytotoxicity [33,96]. A study by Wilk et al., identified a further three exhaustion markers (LAG3, PDCD1 and HAVCR2) on NK cells from COVID-19 patients, indicating that an exhausted NK cell phenotype may well be induced by SARS-CoV-2 infection [97]. Future studies will fully elucidate the role of exhaustion markers on NK function in COVID-19. A higher neutrophil to lymphocyte ratio was also observed, indicating systemic inflammation and infection (Figure 2) [95]. Gene expression analysis has shown that various genes specific to NK function and maturity are downregulated in COVID-19 [97]—FCGR3A and FGFBP2 in particular are associated with cytotoxic CD56^DIM^ cells [98]. All these findings suggest that a more inflammatory NK cell phenotype is present in SARS-CoV-2infection.

COVID-19 patients display high levels of inflammatory cytokines and chemokines (IL-1a/β, IP-10, MCP-1), with severe cases showing elevation in TNFα, IL-1, IL-6, IL-18, IL-8, IL-10, MCP-1 and MIP-1A, leading to severe pulmonary tissue damage [84,86,95,99]. Besides its inflammatory role, IL-1 has also been linked to the expression of thromboxane-A2 in COVID-19 patients, resulting in increased platelet activation and aggregation, which mediate thrombus formation [100,101]. NK cells are likely play a significant role in this cytokine induced damage. Firstly the chemokines MCP-1 and IP-10 recruit NK cells to inflamed tissues, particularly the lungs [102], effector functions of these NK cells are however blunted and skewed towards an inflammatory phenotype. It has been suggested that NK-cell-produced IFN-γ and TNF-α may be functionally linked to NK cell cytolysis through NF-κB-dependent upregulation of ICAM-1 expression in target cells [103]. The decreased number of NK cells combined with the reduced levels of IFN-γ and TNF-α induced through NKG2A expression, may lead to decreased NK cell cytolytic function in SARS-CoV-2. Lastly, IL-6 and IL-10 are a prominent feature of SARS-CoV-2 infection [84,86,87,95,104], and both have the capacity to reduce NK cell cytotoxicity [105,106]. IL-6 has been shown to directly reduce the expression of perforin and granzyme B [105], while IL-10 has been shown to be negatively correlated with NK cell cytotoxicity, through a reduction in IFN-γ and IL-2 expression [106,107]. In viral infection, defective cytotoxicity leads to the accumulation of antigenic stimuli, perpetuating inflammation and in that triggering tissue damage (Table 1). Helper T cells, regulatory T cells and memory T cells are all diminished in severe COVID-19 cases, though Th1 and Th2 cytokine levels remain high [84,95]. Combined, these factors indicate that diminished NK cell cytotoxicity and immune regulation lead to a critical inflammatory phenotype in SARS-CoV-2 infection [108]. Interestingly, despite the overall decrease in NK cell subsets in COVID-19, one study highlighted an increase in CXCR3+ NK cells in an individual with severe disease [109]. CXCR3+ NK cells are more common in the CD56^BRIGHT^ subpopulation, indicating that the balance of NK cell subsets may be skewed towards inflammation, rather than cytotoxicity. Indeed, Mazzoni et al., found an inverse correlation between serum levels of IL-6 and the frequency of NK cells expressing Granzyme A, particularly in intensive care unit (ICU) patients [110].

Little is known about the role of NK cell activating receptors in SARS-CoV-2 infection. In other respiratory viruses such as RSV, Influenza and adenovirus, various mechanisms of inhibition have been described [111,112,113,114,115,116]. NKG2D has broad activation ability through a myriad of ligands, and reduction of NKG2D expression and function allows for viral escape, leading to increased pro-inflammatory cytokine expression as well as increased lung pathology [114]. In RSV, an increase in soluble MICA inhibits NKG2D activity [116], whilst in human cytomegalovirus this is achieved through early expression of type 1 IFN and IL-12 [117]. Influenza virus neuramindase protein allows influenza virus to evade both NKp46 and NKp44 recognition, significantly reducing the killing of infected cells [111]. NKp46 has been shown to be the most predominant cytotoxicity receptor in the nasal lavage of patients infected with respiratory viruses [115]. Shifts in the NKp46 splice variants was observed, with functional differences apparent between variants, the NKp46 domain 1-negative isoform proving to be functionally more active. Additionally it was observed that cytokines modulate the NKp46 splice variant expression profile [115].There is some evidence that lower expression of NKG2C may lead to more severe SARS-CoV-2 outcomes [118], though this is preliminary and much more research is required to understand the effect of SARS-CoV-2 on NK cells’ activating receptors.

Numerous clinical trials are investigating treatment strategies that directly affect NK effector cell function [119,120]. The immunosuppressive drugs, Tocilizumab and Anakinra [34], block IL-6 and IL-1 respectively leading to increased expression of perforin, granzyme A/B and IFNγ [105,121]. Tocilizimab has been shown to restore NK cell cytotoxic activity in 4/5 COVID-19 ICU patients [110]. CYNK-001 is a cryopreserved allogeneic NK cell therapy being developed from placental hematopoietic stem cells [122], these cells express NK activating receptors NKG2D, DNAM-1 and cytotoxicity receptors NKp30, NKp44 and NKp46, they also express both perforin and granzyme B [122]. Another NK cell therapy is the IL-15 superagonist- and granulocyte-macrophage colony-stimulating factor (GM-CSF) neutralizing scFv-secreting NKG2D-ACE2 CAR-NK derived from cord blood. This treatment blocks SARS-CoV-2 infection of ACE2 presenting cells whilst upregulating NK cell cytotoxicity [123]. Treatments such as hydroxychloroquine (HCQ) [124,125] and intravenous immunoglobulin (IVIG) [126] may indirectly improve NK cell function through reduction of T cell activation and the introduction of functional immune cells. HCQ in particular reduces in the production of IL-1β, IL-6 and TNFα [127], thereby decreasing IFNγ release by CD56^BRIGHT^ NK cells, dampening overactive inflammatory responses. Several trials are also investigation the use of oxygen therapy in the early treatment of SARS-CoV-2 infection, as previous studies have shown that this treatment effectively decreases inflammation, reducing IL-12 and TNF-α, and increasing IL10 [128,129]. Numerous trials are investigating the possibility that the Bacille Calmette-Guérin (BCG) vaccine, used to protect young children against tuberculosis (*Mycobacterium tuberculosis*), may offer heterologous protection against SARS-CoV-2 through trained immunity [130]. BCG vaccination may lead to epigenetic changes in monocyte and NK cell populations, resulting in an enhanced immune response to subsequent infections, particularly through increased expression of IL-1β, TNF and IL-6 [131].

The flavonoids Quercetin and Luteolin, have been shown to reduce IL-6 expression in Mast cells, and may be a safer alternative to corticosteroid treatment [132,133]. The role of anti-inflammatory cytokines should not be overlooked, although human trials have yet to be conducted, IL-37 has been shown to suppress systemic inflammation in rheumatoid arthritis [134] and Influenza [135] mouse models. Both IL-37 and IL-38 have been suggested as potential COVID-19 therapeutics [136,137]. A final consideration in the goal to improve NK cell function in COVID-19, is exercise. Physical inactivity has been shown to reduce NK cell activity and IFN-γ expression [138]. Isolation and lockdown strategies to curb the spread of SARS-CoV-2 inadvertently leads to many individuals following a more inactive lifestyle. Reinforcing the importance of physical activity, particularly in high-risk individuals may be of benefit to COVID-19 outcomes.

## 5. Conclusions

Natural killer cells play a central role in maintaining immune homeostasis, a critical requirement when facing the challenge of a novel pathogen. SARS-CoV-2 infection has been shown to impede NK cell function, thus disrupting this vital balance. With factors such as ageing and other comorbidities, leading to further skewing, the current trials investigating drugs and biologicals, which improve NK cell function, may prove to be monumental in our fight against COVID-19.

## Figures and Tables

**Table 1:** Summary of published findings for NK cell dysregulation in disease and infection.

Condition	Findings	Reference
Ageing	NK cell production and proliferation is reduced.	[12,14,15]
	Higher frequency of mature NK cells.	
	NK cell cytotoxicity is reduced and pro-inflammatory cytokines increased.	
	Tissue damage and necrosis are increased due to reduced expression of NKp46+ resulting in decreased neutrophil apoptosis by NK cells and enhancement of neutrophil necrosis.	
Atherosclerosis	Increased frequency of CD56^BRIGHT^ NK cells leads to tissue damage and inflammation due to an increased cytokine production.	[20,21]
	Increased expression of MICA/B, results in elevated activation of natural cytotoxicity receptor NKG2D, increasing NK cell activity.	
Autoimmunity	SLE; Reduced number of cytotoxic NK cells. Anti-NKG2A antibodies result is dysregulated self-nonself-recognition.	[22,23,24,25,26]
	JIA; Reduction in both cytolytic and inflammatory NK cell activity. Patients can develop MAS	
	MS; Reduction in CD56^DIM^ NK cells results in decreased cytolytic activity and impaired regulation of CD4* T cells, resulting in increased inflammatory responses.	
Hematologic	Reduced CD58 expression leads to reduced NK cell activation.	[27,28,29,30]
Malignancy	Increased CD47 and IDO expression suppress NK cells activity.	
	Increased CXCR1 expression leads to increased CD56^DIM^ NK cell activity resulting in necrosis, apoptosis and organ failure.	
RSV	Decreased expression of NKG2D and NKp44 reducing cytolytic activity.	[31,32]
	Increased IFN-γ expression inducing pro-inflammatory response.	
COVID-19	NK cells display exhausted phenotype and are reduced in number.	[33,34,35]
	Reduced cytotoxicity through increased NKG2A expression.	
	Increased production of inflammatory cytokines and chemokines.	

Definition of abbreviations: NK = natural killer; MICA/B = major histocompatibility complex (MHC) class I chain-related protein A and B; SLE = systemic lupus erythematosus; JIA = juvenile idiopathic arthritis: MAS = macrophage activation syndrome; MS = multiple sclerosis; IDO = Indoleamine 2,3-dioxygenase.

## Abbreviations

ARDS	Acute respiratory distress syndrome
HLH	Hemophagocytic lymphohistiocytosis
NK	Natural killer
MHC	Major histocompatibility complex
KIR	Killer cell immunoglobulin receptors
IFN	Interferon
TLR	Toll-like receptor
ADCC	Antibody-dependent cellular cytotoxicity
TNF	Tumor necrosis factor
MIP	Macrophage inflammatory protein
DC	Dendritic cells
NCR	Natural cytotoxicity receptors
LDL	Low density lipoprotein
Th1	T helper cell 1
SLE	Systemic lupus erythematosus
HLA	Human leukocyte antigen
IL	Interleukin
PD-1	Programmed cell death protein
MAS	Macrophage activation syndrome
RSV	Respiratory syncytial virus
SARS	Severe acquired respiratory syndrome
ACE2	Angiotensin-converting enzyme 2
ANKL	Aggressive natural killer leukemia
JIA	Juvenile idiopathic arthritis
MS	Multiple sclerosis
